# Meal frequency patterns and glycemic properties of maternal diet in relation to preterm delivery: Results from a large prospective cohort study

**DOI:** 10.1371/journal.pone.0172896

**Published:** 2017-03-01

**Authors:** Linda Englund-Ögge, Bryndis Eva Birgisdottir, Verena Sengpiel, Anne Lise Brantsæter, Margareta Haugen, Ronny Myhre, Helle Margrete Meltzer, Bo Jacobsson

**Affiliations:** 1 Department of Obstetrics and Gynecology, Sahlgrenska University Hospital, Gothenburg, Sweden; 2 Department of Environmental Exposure and Epidemiology, Domain of Infection Control and Environmental Health, Oslo, Norway; 3 Unit for Nutrition Research, National University Hospital, Faculty of Food Science and Nutrition, University of Iceland, Reykjavik, Iceland; 4 Department of Genetics and Bioinformatics, Domain of Health Data and Digitalization, Institute of Public Health, Oslo, Norway; 5 Department of Obstetrics and Gynecology, Institute of Clinical Sciences, Sahlgrenska Academy, Gothenburg University, Gothenburg, Sweden; Fondazione Toscana Gabriele Monasterio, ITALY

## Abstract

**Background:**

Dietary habits are linked to high maternal glucose levels, associated with preterm delivery. The aim of this study was to examine the associations between meal frequency and glycemic properties of maternal diet in relation to preterm delivery.

**Methods:**

This prospective cohort study included 66,000 women from the Norwegian Mother and Child Cohort Study (MoBa). Meal frequency and food intake data were obtained from a validated food frequency questionnaire during mid-pregnancy. Principal component factor analysis was used with a data-driven approach, and three meal frequency patterns were identified: “snack meal”, “main meal”, and “evening meal”. Pattern scores were ranked in quartiles. Glycemic index and glycemic load were estimated from table values. Intakes of carbohydrates, added sugar, and fiber were reported in grams per day and divided into quartiles. Gestational age was obtained from the Medical Birth Registry of Norway. Preterm delivery was defined as birth at <37 gestational weeks. A Cox regression model was used to assess associations with preterm delivery.

**Results:**

After adjustments, the “main meal” pattern was associated with a reduced risk of preterm delivery, with hazard ratios (HRs) of 0.89 (95% confidence interval (CI): 0.80, 0.98) and 0.90 (95% CI: 0.81, 0.99) for the third and fourth quartiles, respectively, and *p* for trend of 0.028. This was mainly attributed to the group of women with BMI ≥25 kg/m^2^, with HRs of 0.87 (95% CI: 0.79, 0.96) and 0.89 (95% CI: 0.80, 0.98) for the third and fourth quartiles, respectively, and *p* for trend of 0.010. There was no association between glycemic index, glycemic load, carbohydrates, added sugar, fiber, or the remaining meal frequency patterns and preterm delivery.

**Conclusion:**

Regular consumption of main meals (breakfast, lunch, dinner) was associated with a lower risk of preterm delivery. Diet should be further studied as potential contributing factors for preterm delivery.

## Introduction

Preterm delivery is a condition associated with serious short- and long-term neonatal complications [1,2], as well as neonatal death. It is defined as giving birth before gestational week 37 and is either spontaneous or iatrogenic [3]. Although the neonatal survival rate has increased overall, mainly because of improvements in neonatology, [4] the incidence rates are constant. In the majority of cases, the causes of spontaneous preterm delivery remain unknown [5].

The importance of meal frequency in human health and in the prevention of disease has been debated [6–8]. Recommendations regarding meal regularity and frequency in pregnancy may be important [9], since, in animal studies, longer food withdrawal was shown to increase the production of prostaglandins [10] and the contractions of the uterus [11]. Irregular meals are also correlated to detrimental effects on postprandial glucose concentrations and increased insulin resistance [12]. High plasma glucose levels increase prostaglandin response in animal studies [13], which have been linked to preterm delivery [14]. Likewise, this association is also observed in humans, since elevated blood glucose levels are associated with preterm delivery among women with gestational diabetes [15]. Preterm delivery is also more prevalent in pregnant women with elevated blood sugar levels *below* the diagnosis criteria of diabetes [16]. Pregnancy itself is a state of insulin resistance [17], and especially high postprandial plasma glucose levels lead to high insulin levels, which can have multiple negative long-term effects, e.g., increased oxidative stress with endothelial dysfunction, an unfavorable composition of plasma lipids that is related to vascular damage, and an increase in clotting factors [18]. Current guidelines lack recommendations regarding meal frequency in pregnant women [19].

However, studying meal frequency patterns can be complex, as the combination of meals can vary interminably in populations. One way to overcome this issue partly is to use principal component analysis, a method for assessing the overall combinations and for detecting structures in the study population without predefined patterns. This approach was recently used in another study [20], but we used this approach as a tool to assess the association between meal frequency patterns and preterm delivery.

Not only meal frequency, but also the quality of diet and especially the glycemic properties of food affect the postprandial plasma glucose response [21]. An association between dietary carbohydrate content and quality and preterm delivery has been suggested [22]. A study from the US found that high dietary glycemic index (GI), a factor presumed to describe the effect of the diet on postprandial glucose, was associated with a higher risk of preterm delivery [22]. Another study found that dietary glycemic load (GL), an arithmetic variable relating GI to the amount of consumed carbohydrates, was associated with preterm delivery among overweight and obese women [23].

The main objectives of this study were as follows: firstly, to examine the association between meal frequency and preterm delivery; secondly, to investigate whether carbohydrate content and quality, expressed as glycemic index (GI), glycemic load (GL), and estimated intakes of carbohydrates, added sugar, and dietary fiber, were associated with preterm delivery.

## Methods

### Population and study design

This study was based on data from the Norwegian Mother and Child Cohort study (MoBa), a prospective population-based pregnancy cohort study conducted by the Norwegian Institute of Public Health [24]. Participants were recruited from all over Norway from 1999 to 2008. Consent to participate was given in 40.6% of the pregnancies. *The cohort now includes 114,500 children, 95,200 mothers, and 75,200 fathers* [25]. Participants were invited by post, prior to the free routine ultrasound examination during gestational weeks 17–18. Women were followed up with questionnaires during pregnancy and the data in this study were derived from two questionnaires, answered during gestational weeks 17 and 22, respectively. In questionnaire 1 (Q1), women were asked to report information about lifestyle, background, illness, and health-related factors. Questionnaire 2 was a semi-quantitative food frequency questionnaire (FFQ), in which women were asked to report their daily intake of food and beverages from the beginning of the current pregnancy. Pregnancy outcomes were recorded in the Medical Birth Registry of Norway and were linked to the MoBa database [26]. The Regional Committee for Ethics in Medical Research (REK/S-06075) and the Data Inspectorate in Norway approved the study. Written informed consent was obtained from all participants.

**Fig 1:**
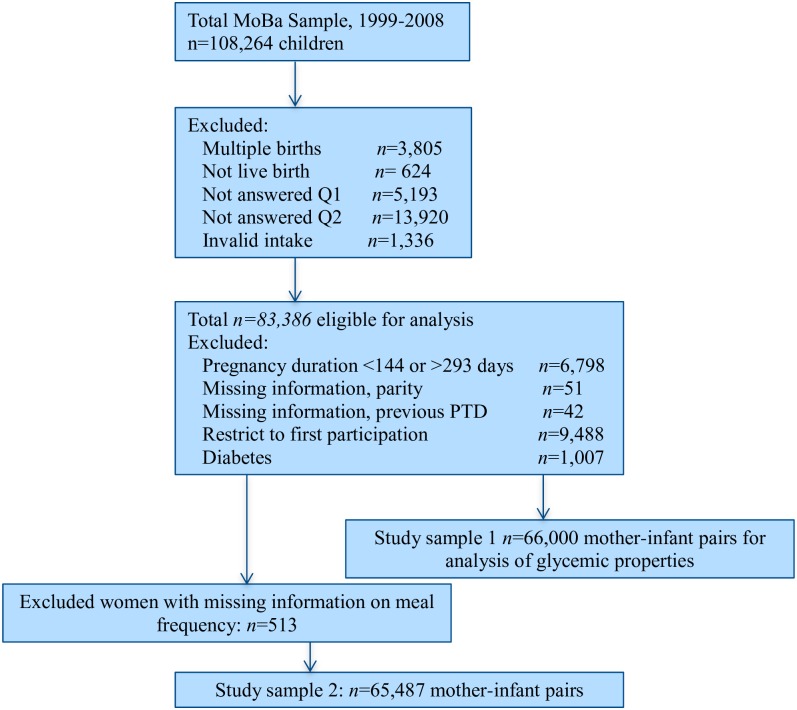
Flow chart showing selection of study participants. Q1 denotes the first prenatal questionnaire, Q2 denotes the MoBa food frequency questionnaire used from 2002.

Data files released for research in 2010 (version 5) were used in this study (n = 108,264 children). To be eligible for the study (Fig 1), women had to have delivered a live singleton baby, to have responded to the general questionnaire (Q1) and the FFQ (Q2), and to have a valid energy intake (4.5–20 MJ) (n = 83,386). Furthermore, gestational age between 22 + 0 and 41 + 6 weeks, and information about any previous preterm delivery and parity were required for inclusion. To avoid multiple dependent observations in the analyses, only the first pregnancy of each woman enrolled in the cohort was included. Based on an a-priori decision, we excluded women with a history of diabetes as well as those diagnosed during pregnancy, since dietary modification is an integral part of diabetes treatment, which left a study sample of 66,000 mother-infant pairs. In the analyses of meal frequency patterns, only women who reported eating at least one meal a day were included, resulting in a study sample of n = 65,487.

### Dietary information

The MoBa FFQ (downloadable at http://www.fhi.no/dokumenter/011fbd699d.pdf) was introduced and collected from March 2002 onward. The FFQ is a semi-quantitative, validated questionnaire designed to capture information on dietary intake from the start of the current pregnancy [27]. In the FFQ, women are asked to report how many times they consumed the following eight meals: breakfast, morning snack, lunch, afternoon snack, dinner, evening snack, supper, and night meal, with eight response alternatives ranging from zero to seven times weekly. The FFQ covers 255 foods and beverages, and a validation study showed that it is a valid tool for ranking pregnant women’s dietary intake according to high and low consumption of food, energy, and nutrients [28]. In the present study, the FFQ was optically read and food frequencies were converted into daily intakes, reported in g/day. The Norwegian Food Composition Table and FoodCalc were used to calculate energy and nutrient intakes [29]. Total carbohydrates, added sugar, and dietary fiber were reported in g/day. A GI database was constructed based on the FFQ, using values from international glucose reference tables [30]. The GI of a food item is a value based on the effect that 50 g of carbohydrates from the food item have on postprandial blood sugar over a two-hour period in an experimental setting, compared to the same amount of carbohydrates in the form of glucose. Food, yielding a large area under the curve because of its rapid absorption, such as fruit juice, has a high GI, while food yielding a smaller area under the curve, such as an apple, has a low GI. Food containing no or a negligible amount of carbohydrates were not included in the GI database (e.g., meat, fish, fats, and eggs). The total dietary GL for each participant was calculated by adding the GIs of each food item, multiplied by the amount of carbohydrates in the food items. The total dietary GI was calculated from the total dietary GL, divided by the total amount of carbohydrates consumed by each participant [31].

### Extraction of meal frequency patterns

We used principal component factor analysis with orthogonal (varimax) rotation to extract meal frequency patterns, with the weekly frequencies of the eight meals as input variables. This data-driven technique correlates variables and identifies new linear components [32]. The components (meal frequency patterns) reflect the combinations of meals consumed by the women. The number of retained components was based on a scree plot (S1 Fig) that shows the proportion of the variance in total consumption of meals [33]. Meals with factor loadings >± 0.50 were considered important for the interpretability of the characteristics of each meal frequency pattern. We named the components (the meal frequency patterns) according to the characteristics of the meals with the highest factor loadings.

### Covariates

Eleven covariates were chosen because of their known association with preterm delivery. Information about maternal age at delivery and previous preterm delivery was collected from the Medical Birth Registry of Norway. Maternal age was used as a continuous variable, except in the case of descriptive statistics, in which it was divided into four groups (<20 years, 20–29 years, 30–39 years, and ≥40 years). Data on any history of previous preterm delivery was analyzed as a dichotomous variable (yes or no). Pre-pregnancy weight and height, from which BMI was calculated, was collected from Q1. Only the women who reported weight in the range of 35–180 kg and height above 1.40 m were included. BMI was divided into four categories (<18.5 kg/m^2^, 18.5–24.9 kg/m^2^, 25–29.9 kg/m^2^, ≥30 kg/m^2^) and analyzed as categorical data. Height was ranked into quartiles (≤1.64 cm, 1.65–1.68 cm, 1.69–1.72 cm, ≥1.73 cm). Information about marital status was collected from Q1 and categorized as either living alone or cohabiting. Parity was analyzed as dichotomous data (nulliparous or multiparous) and the information was collected from both Q1 and the Medical Birth Registry of Norway. Smoking status was categorized as non-smokers, occasional, or daily smokers. Maternal education was divided into ≤12 years, 13–16 years, or ≥17 years. Total energy intake was analyzed as a continuous variable, except for the description of the glycemic properties by energy intake, for which it was ranked into quartiles. Information about household income was obtained from Q1 and divided into three categories (both partners <300,000 NOK per year, participant or partner ≥300,000 NOK per year, and both partners ≥300,000 NOK per year). Alcohol intake during pregnancy was analyzed as dichotomous data (yes or no), as was information about first-trimester nausea, in vitro fertilization (IVF), and working regular or irregular hours, as reported in Q1. In a previous publication we found that overall dietary patterns were associated with preterm delivery [34]. Associations between meal frequency patterns and preterm delivery could possibly be confounded by women’s overall dietary quality. Dietary fiber intake is a good indicator of an overall healthy diet [12]. Therefore, we included dietary fiber among the confounding variables in a sub-analysis. We also adjusted analysis according to maternal physical activity, as a previous MoBa study showed that exercise performed during pregnancy reduced the prevalence of preterm delivery [35]. In gestational week 17, women reported their physical activity and these data were categorized for analysis (none, less than weekly, 1–2 times weekly, and >2 times weekly).

### Preterm delivery

The primary outcome was preterm delivery, defined as delivery before gestational week 37 + 0. Gestational age was calculated using ultrasound measurements taken during gestational weeks 17–18. Information about gestational age at birth was obtained from the Medical Birth Registry of Norway [26]. In 1,154 cases (1.7%), ultrasound information was missing, and therefore, gestational age was calculated based on the date of the last menstruation. Preterm delivery was examined in subgroups based on onset (spontaneous or iatrogenic) and gestational age. Iatrogenic delivery was defined as induced vaginal or primary caesarean on maternal or fetal indication, and gestational age at the time of delivery was divided into late (34 + 0–36 + 6 weeks), moderate (32 + 0–33 + 6 weeks), or early (22 + 0–31 + 6 weeks) preterm.

### Statistical methods

PASW Statistics software, version 19 for Windows Statistical (SPSS Inc., IBM Company, Chicago, Ill., USA) was used for the statistical analysis. For assessment of normality, we used the Kolmogorov-Smirnov test. In addition, we visually inspected histograms and curves together with descriptive statistics. We used the Mann-Whitney test as a non-parametric test to compare means in non-normally distributed data when comparing two groups. The Kruskal-Wallis test was used for comparing means in non-normally distributed data for more than two groups. Continuous data were described as mean ± standard deviation (SD). The Analysis of Variance (ANOVA) test was used to assess statistical differences between groups of continuous data with normal distribution. Post-hoc analysis with Bonferroni correction was applied to compare the main meals factor scores among the maternal pre-pregnancy BMI categories.

The meal frequency patterns were extracted using principal component factor analysis in SPSS. This is an exploratory data analysis that uses correlation matrix of the variables to identify patterns, with the main goal of simplifying and reducing the data [32]. To test the appropriateness of data for the principal component factor analysis, we used the Bartlett´s Test of Sphericity and the Kaiser-Meyer-Olkin measure of sampling adequacy. Bartlett’s Test of Sphericity was significant (*p* <0.01) and the Kaiser-Mayer-Olkin test was 0.5, which is borderline acceptable. The factor loading coefficients of the respective meals were calculated and presented, as well as the cumulative variance of the three meal frequency patterns. Meal frequency pattern scores were created from the factor loadings, and rotated in the analysis. Factor loadings were standardized before being used in the model. SPSS combines principal component analysis PCA and factor analysis to create a principal component factor analysis model. Meal frequency pattern scores were divided into quartiles. We used a Cox regression model with gestational age in days as the time variable and the event defined as preterm delivery. Deliveries after the preterm period were censored. Data is presented as crude and adjusted data. The Cox regression models were adjusted for the following confounders: maternal age, pre-pregnancy BMI, height, parity, total energy intake, maternal education, marital status, smoking status, income, and previous preterm delivery. In additional analyses, we also adjusted for dietary fiber intake as a proxy for overall healthy eating, alcohol intake, and nausea during the first trimester, as well as working irregular hours and physical activity as reported at gestational week 17. Analyses were stratified into subgroups of BMI (<25 kg/m^2^ or ≥25 kg/m^2^) and maternal age (<35 or ≥35 years), since these two groups are generally more insulin resistant [36,37] and might respond to differently to high postprandial levels of glucose, with subsequently different outcomes. We also stratified the material based on preterm premature ruptures of membranes (PPROM), because previous studies found an association between meal frequency and PPROM [38]. Results were presented as hazard ratios (HRs) with 95% confidence intervals (95% CIs). All meal frequency patterns were entered into the same model. All *p* values were 2 sided and <0.05 was considered statistically significant. Simple linear regression analyses were used to test for a trend in the means (i.e., *p*-trend).

We also used a Cox regression model to assess the associations between the glycemic properties (GI, GL, total carbohydrates, added sugar, and dietary fiber) of the maternal diet and preterm delivery in terms of days of gestation. Each glycemic property was analyzed separately, both as crude and adjusted data. Models were adjusted for maternal age, pre-pregnancy BMI, height, parity, total energy intake, maternal education, marital status, smoking status, income, and previous preterm delivery. In a separate analysis, GI, GL, total carbohydrates, and added sugar were also adjusted for dietary fiber intake. *P* values for linear trend were obtained by incorporating each variable as a linear term in the Cox regression models. Since multiple testing was done, a modified Bonferroni correction was performed for variables with a *p* value that fell below the significance level, significant only in one of the analyses in which the sum of all *p* values for that specific variable was divided by the total number of observations.

## Results

### Preterm delivery

In the study sample of 66,000 pregnant women, preterm delivery occurred in 3,505 (5.3%) cases, of which 2,003 (3.1%) and 1,414 (2.2%) underwent spontaneous and iatrogenic deliveries, respectively. Information about the delivery start was missing in 88 (0.13%) cases. When divided according to gestational week, 469 (0.7%) were early preterm deliveries, 478 (0.7%) were moderately preterm, and 2,558 (3.9%) were late preterm. PPROM was diagnosed in 932 cases (1.4%).

### Meal frequency patterns

The scree plot shows the three meal frequency patterns with eigenvalues >1 (S1 Fig). These three patterns accounted for 52% of the variation in reported meal frequencies (Table 1). The first pattern had an eigenvalue of 1.6, and explained 19% of the variance in reported meal frequencies. This pattern had high factor loadings for snack meals in the morning, afternoon, and evening, and it was named the “snack meal” pattern. The second pattern had high factor loadings for breakfast, lunch, and dinner, and it was named the “main meal” pattern. This pattern had an eigenvalue of 1.5 and explained 19% of the variance. The third pattern had high factor loadings for supper, night meal, and morning snack, with an eigenvalue of 1.2. This pattern was named the “evening meal” pattern. The total cumulative variance for all three patterns was 52%.

**Table 1:** Structures of the three orthogonally rotated factors (meal frequency patterns) identified using principal component factor analysis in 65,487 women in the Mother and Child Cohort Study (MoBa)

Meal^1^	“Snack meal pattern”	“Main meal pattern”	“Evening meal pattern”
**Breakfast**		**0.69**	
**Morning snack**	**0.62**^2^		
**Lunch**		**0.68**	
**Afternoon snack**	**0.74**		
**Dinner**		**0.63**	
**Evening snack**	**0.73**		
**Supper**			**0.82**
**Night meal**			**0.53**
**Cumulative variance, %**^3^	**19**	**38**	**52**

^1^Designated meal frequency pattern

^2^Factor loading coefficient

^3^Percentage of variance in total meal frequency explained by the patterns

The “main meal” frequency pattern includes breakfast, lunch, and dinner. Those with high adherence to this pattern (i.e. ranked in the upper quartile) consumed a median number of four meals per day, four main meals or three main meals and one snack, as compared to one main meal among those in the lowest quartile of this pattern. The meal frequency scores were weakly correlated with the GI and GL properties; the highest correlations (Spearman’s rho) were r = 0.12 (p<0.001) between the “evening meal” and GL, and r = 0.44 (p<0.001) between the “main meal” and GL.

### Maternal characteristics in relation to meal frequency patterns and glycemic properties

Upon investigation of meal frequency patterns in relation to maternal characteristics, increased maternal age was significantly associated with higher adherence to the “main meal” frequency pattern, and younger women was associated with higher adherence to the “evening meal” frequency pattern (S1 Table). Women in the lowest educational group (<12 years in school) had higher scores for the “evening meal” pattern than those of the women with higher education. Normal weight women had higher scores for the “main meal” pattern than those of the women in other groups. Overweight and obese women had lower scores for the main meal pattern, indicating a lower adherence to this pattern. According to the Bonferroni post hoc test, all BMI groups were significantly different from each other, except for the underweight group compared to the obese group when analyzing the main meal pattern. Women who reported daily smoking had higher scores on the “evening meal” pattern than non-smokers. Cohabiting women reported the highest adherence to the “main meal” frequency pattern, while single women had similar meal pattern scores for the “evening meal” frequency pattern. Women in the highest income groups were more frequently eating according to the “main meal” frequency pattern (S1 Table).

All of the glycemic properties, GI, GL, and the daily intake of total carbohydrates, added sugar, and dietary fiber, also differed according to maternal characteristics (S2 Table). Higher maternal age was associated with lower GL, total carbohydrates, added sugar, and higher fiber intake. Higher maternal educational level was associated with lower GL, total carbohydrates, added sugar, and with higher dietary fiber intake. GI was also lower in women with higher education, but the association was non-significant (p = 0.161). Women in the highest BMI groups had lower GL, carbohydrate, and fiber intake. They also reported low intake of added sugar. Daily smokers showed a higher intake of added sugar and total carbohydrates and a lower intake of dietary fiber than did non-smokers. Women with the highest household income had the lowest GI, GL, and total carbohydrate and added sugar intake.

### Meal frequency patterns in relation to preterm delivery

Women in the two highest quartiles of the “main meal” pattern (Table 2) had the lowest risk of preterm delivery. This was seen in both unadjusted and adjusted analysis, with adjusted HRs of 0.89 (95% CI: 0.80, 0.98) and 0.90 (95% CI: 0.81, 0.99) for the third and fourth quartiles, respectively, with *p* for trend of 0.028. Adjusting for dietary fiber intake as an indicator of an overall healthy diet did not change the results significantly. The HRs for the third and fourth quartiles were 0.89 (95% CI: 0.79, 0.99) and 0.88 (0.78, 0.99), respectively, with a p for trend = 0.046.

**Table 2:** Associations between meal frequency patterns and preterm delivery in 65,487 pregnant women in the Norwegian Mother and Child Cohort Study (MoBa)

Meal frequency pattern^1^	PTD n (%^2^)	Model 1^3^	Model 2^4^
		HR (95% CI)	HR (95% CI)
	3,473 (5.3)		
**“Snack meal” pattern**			
**Quartile 1**	875 (1.3)	1	1
**Quartile 2**	842 (1.3)	0.98 (0.89, 1.07)	0.98 (0.89, 1.07)
**Quartile 3**	873 (1.3)	1.01 (0.92, 1.11)	1.00 (0.91, 1.10)
**Quartile 4**	883 (1.3)	1.04 (0.94, 1.14)	1.00 (0.90, 1.10)
***p* for trend**^5^		*0*.*566*	*0*.*821*
**“Main meal” pattern**			
**Quartile 1**	979 (1.5)	1	1
**Quartile 2**	905 (1.4)	0.93 (0.85, 1.02)	0.99 (0.90, 1.08)
**Quartile 3**	779 (1.2)	0.80 (0.72, 0.88)	0.89 (0.80, 0.98)
**Quartile 4**	807 (1.2)	0.79 (0.72, 0.87)	0.90 (0.81, 0.99)
***p* for trend**^5^		*<0*.*001*	*0*.*028*
**“Evening meal” pattern**			
**Quartile 1**	829 (1.3)	1	1
**Quartile 2**	879 (1.3)	1.08 (0.98, 1.19)	1.06 (0.96, 1.17)
**Quartile 3**	892 (1.4)	1.14 (1.02, 1.26)	1.10 (0.99, 1.23)
**Quartile 4**	873 (1.3)	1.06 (0.96, 1.17)	1.03 (0.93, 1.14)
***p* for trend**^5^		*0*.*083*	*0*.*403*

^1^ Meal frequency patterns were created by multiplying the factor loading by the corresponding standardized value for the intake of each meal and then adding them together. Scores were analyzed as categorical data and divided into quartiles.

^2^ Percentage of preterm delivery in each quartile

^3^ Unadjusted hazard ratio, but adjusted for the other meal frequency patterns

^4^ Hazard ratios adjusted for maternal age, pre-pregnancy BMI, height, parity, total energy intake, maternal education, marital status, smoking, income, previous preterm delivery, and the other meal frequency patterns

^5^ *p* values for linear trend were obtained by incorporating the variable as a linear term in Cox regression models

Analysis of data according to gestational age (Table 3) showed that high adherence to the “main meal” pattern was associated with a reduced risk of late preterm delivery, with adjusted HR 0.88 (95% CI: 0.78, 0.99) for the highest versus the lowest quartile, but a non-significant *p* for trend, 0.061. The HR was the same after adding adjusting for dietary fiber intake. The association between meal frequency and early preterm delivery followed a similar pattern but was not significant.

**Table 3:** Associations between meal frequency patterns and preterm delivery in 65,487 pregnant women in the Norwegian Mother and Child Cohort Study (MoBa)

Meal frequency pattern^1^	PTD n (%)^2^	Early PTD Model 1^3^	Early PTD Model 2^4^	PTD n (%)^2^	Moderately PTD Model ^3^	Moderately PTD Model 2^4^	PTD n (%)^2^	Late PTD Model 1^3^	Late PTD Model 2^4^
		HR (95% CI)	HR (95% CI)		HR (95% CI) ^4^	HR (95% CI)		HR (95% CI)	HR (95% CI)
**“Snack meal” pattern**									
**Quartile 1**	118 (0.2)	1	1	112 (0.2)	1	1	644 (1.0)	1	1
**Quartile 2**	108 (0.2)	0.95 (0.73, 1.23)	0.95 (0.73, 1.24)	114 (0.2)	1.00 (0.77, 1.29)	1.03 (0.79, 1.35)	620 (1.0)	0.98 (0.88, 1.09)	0.97 (0.87, 1.08)
**Quartile 3**	118 (0.2)	1.05 (0.81, 1.36)	1.03 (0.79, 1.34)	128 (0.2)	1.09 (0.84, 1.41)	1.12 (0.86, 1.46)	627 (1.0)	0.99 (0.89, 1.11)	0.97 (0.87, 1.09)
**Quartile 4**	120 (0.2)	1.08 (0.84, 1.40)	1.02 (0.79, 1.34)	120 (0.2)	1.05 (0.81, 1.36)	1.05 (0.80, 1.38)	643 (1.0)	1.03 (0.92, 1.15)	0.98 (0.88, 1.10)
***p* for trend**^5^		*0*.*642*	*0*.*992*		*0*.*449*	*0*.*420*		*0*.*881*	*0*.*539*
**“Main meal” pattern**									
**Quartile 1**	140 (0.2)	1	1	125 (0.2)	1	1	714 (1.1)	1	1
**Quartile 2**	122 (0.2)	0.88 (0.69, 1.12)	0.94 (0.73, 1.21)	145 (0.2)	1.16 (0.91, 1.78)	1.29 (1.01, 1.66)	641 (1.0)	0.90 (0.80, 1.00)	0.95 (0.85, 1.06)
**Quartile 3**	100 (0.2)	0.73 (0.56, 0.94)	0.83 (0.63, 1.08)	93 (0.1)	0.74 (0.56, 0.97)	0.89 (0.67, 1.19)	586 (0.9)	0.82 (0.73, 0.91)	0.89 (0.80, 1.00)
**Quartile 4**	102 (0.2)	0.67 (0.50, 0.86)	0.78 (0.59, 1.03)	112 (0.2)	0.93 (0.71, 1.21)	1.18 (0.89, 1.57)	593 (0.9)	0.79 (0.71, 0.89)	0.88 (0.78, 0.99)
***p* for trend**^5^		*0*.*002*	*0*.*120*		*0*.*046*	*0*.*928*		*<0*.*001*	*0*.*061*
**“Evening meal” pattern**									
**Quartile 1**	99 (0.2)	1	1	122 (0.2)	1	1	608 (0.9)	1	1
**Quartile 2**	119 (0.2)	1.25 (0.95, 1.64)	1.25 (0.95, 1.64)	120 (0.2)	1 (0.77, 1.29)	0.99 (0.76, 1.30)	640 (1.0)	1.07 (0.96, 1.20)	1.04 (0.93, 0.17)
**Quartile 3**	127 (0.2)	1.44 (1.07, 1.92)	1.44 (1.07, 1.92)	106 (0.2)	0.88 (0.66, 0.18)	0.87 (0.64, 0.16)	659 (1.0)	1.14 (0.10, 1.29)	1.10 (0.97, 1.25)
**Quartile 4**	119 (0.2)	1.23 (0.94, 1.62)	1.20 (0.90, 1.60)	127 (0.2)	1.04 (0.80, 1.35)	1.04 (0.79, 1.36)	627 (1.0)	1.04 (0.92, 1.16)	1.00 (0.89, 1.12)
***p* for trend**^5^		*0*.*080*	*0*.*162*		*0*.*760*	*0*.*805*		*0*.*246*	*0*.*786*

^1^ Meal frequency patterns were created by multiplying the factor loading by the corresponding standardized value for the intake of each meal and then adding them together. Scores were analyzed as categorical data and divided into quartiles.

^2^ Percentage of preterm delivery in each quartile

^3^ Unadjusted hazard ratio, but adjusted for the other meal frequency patterns

^4^ Hazard ratios adjusted for maternal age, pre-pregnancy BMI, height, parity, total energy intake, maternal education, marital status, smoking, income, previous preterm delivery, and the other meal frequency patterns

^5^ *p* values for linear trend were obtained by incorporating the variable as a linear term in Cox regression models

After stratification for BMI (Table 4), only the women with a BMI ≥25 kg/m^2^ had a significantly reduced risk of preterm delivery if they ate according to a “main meal frequency pattern”, with adjusted HRs of 0.87 (95% CI: 0.79, 0.96) and 0.89 (95% CI: 0.80, 0.98) for the third and fourth quartiles, respectively, and a *p* for trend of 0.010. When adjusting for dietary fiber intake, HR was 0.84 (0.70, 0.99) for the fourth quartile with a non-significant *p* for trend. Overweight and obese women had negative scores on “all meal patterns” (S1 Table), indicating that on average, overweight and obese women do not have a typical “main meal” pattern. However, as shown in Table 4, the overweight/obese women with higher adherence to the “main meal” pattern (increasing quartiles) did indeed experience benefit.

**Table 4:** Associations between meal frequency patterns and preterm delivery in 65,487 pregnant women in the Norwegian Mother and Child Cohort Study (MoBa), stratified based on BMI

Meal frequency pattern^1^	PTD n (%)^2^	Model 1^3^	Model 2^4^
		HR (95% CI)	HR (95% CI)
**BMI <25**	2,208 (5.0)		
“Snack meal” pattern			
Quartile 1	523 (1.2)	1	1
Quartile 2	514 (1.2)	0.94 (0.83, 1.06)	0.93 (0.82, 1.05)
Quartile 3	563 (1.3)	1.00 (0.86, 1.13)	0.98 (0.87, 1.10)
Quartile 4	608 (1.4)	1.04 (0.92, 1.17)	0.97 (0.86, 1.10)
*p* for trend^5^		*0*.*467*	*0*.*756*
“Main meal” pattern			
Quartile 1	575 (1.3)	1	1
Quartile 2	569 (1.3)	0.94 (0.84, 1.06)	1.00 (0.89, 1.12)
Quartile 3	516 (1.2)	0.81 (0.72, 0.91)	0.89 (0.78, 1.01)
Quartile 4	548 (1.2)	0.82 (0.72, 0.92)	0.92 (0.81, 1.05)
*p* for trend^5^		*<0*.*001*	*0*.*101*
“Evening meal” pattern			
Quartile 1	516 (1.2)	1	1
Quartile 2	543 (1.2)	0.94 (0.83, 1.06)	1.07 (0.95, 1.22)
Quartile 3	561 (1.3)	1.00 (0.89, 1.13)	1.10 (0.96, 1.26)
Quartile 4	588 (1.3)	1.04 (0.92, 1.17)	1.08 (0.95, 1.22)
*p* for trend^5^		*0*.*016*	*0*.*178*
**BMI ≥25**	1172 (6.0)		
“Snack meal” pattern			
Quartile 1	327 (1.7)	1	1
Quartile 2	305 (1.6)	1.04 (0.89, 1.22)	0.97 (0.88, 1.07)
Quartile 3	290 (1.5)	1.06 (0.90, 1.24)	0.99 (0.90, 1.09)
Quartile 4	250 (1.3)	1.06 (0.90, 1.25)	0.99 (0.89, 1.09)
*p* for trend^5^		*0*.*696*	*0*.*635*
“Main meal” pattern			
Quartile 1	372 (1.9)	1	1
Quartile 2	309 (1.6)	0.90 (0.78, 1.05)	0.98 (0.89, 1.08)
Quartile 3	249 (1.3)	0.82 (0.70, 0.97)	0.87 (0.79, 0.96)
Quartile 4	242 (1.2)	0.80 (0.66, 0.93)	0.89 (0.80, 0.98)
*p* for trend^5^		*0*.*008*	*0*.*010*
“Evening meal” pattern			
Quartile 1	293 (1.5)	1	1
Quartile 2	309 (1.6)	1.025 (0.87, 1.21)	1.06 (0.96, 1.17)
Quartile 3	309 (1.6)	1.14 (0.96, 1.36)	1.10 (0.99, 1.23)
Quartile 4	261 (1.3)	0.93 (0.78, 1.10)	1.03 (0.93, 1.14)
*p* for trend^5^		*0*.*718*	*0*.*389*

^1^ Meal frequency patterns were created by multiplying the factor loading by the corresponding standardized value for the intake of each meal and then adding them together. Scores were analyzed as categorical data and divided into quartiles.

^2^ Percentage of preterm delivery in each quartile

^3^ Unadjusted hazard ratio, but adjusted for the other meal frequency patterns

^4^ Hazard ratios adjusted for maternal age, pre-pregnancy BMI, height, parity, total energy intake, maternal education, marital status, smoking, income, previous preterm delivery, and the other meal frequency patterns

^5^ *p* values for linear trend were obtained by incorporating the variable as a linear term in Cox regression models

The other two meal frequency patterns were not significantly associated with preterm birth, in either the crude or adjusted analyses.

### Glycemic properties in relation to preterm delivery

There were no significant associations between any of the glycemic properties (GI, GL, total carbohydrates, added sugar, or dietary fiber) and overall preterm delivery (S3 Table). Sub-analysis for early preterm, moderately preterm, and late preterm delivery showed that high GL was associated with an increased risk of late preterm delivery, with an adjusted HR of 1.31 (95% CI: 1.06, 1.63) and a *p* for trend of 0.015 for the highest versus the lowest quartile (data not shown). When the study population was stratified based on age (under 35 years or 35 years and above), the highest quartile of dietary fiber intake was associated with a lower risk of preterm delivery, with an adjusted HR of 0.84 (95% CI: 0.73, 0.97) and *p* for trend of 0.017 for women under 35. However, a trend toward higher GL and an increased risk of preterm delivery was found in women aged 35 years or older (data not shown). After adjusting for *p* values according to the Bonferroni correction, none of the above associations was significant, with *p* values of 0.379 and 0.281 for GL and dietary fiber intake, respectively.

No other significant associations were found in the sub-analysis for spontaneous versus iatrogenic preterm delivery or gestational age as a continuous outcome, nor after stratification based on pre-pregnancy BMI or in the sub-analysis for PPROM. Adjusting for nausea in early pregnancy, alcohol intake during pregnancy, IVF pregnancies, or shift work did not alter the results. Additional adjustment for exercise during pregnancy marginally attenuated the results; the HR for the third quartile of the main meal pattern changed from 0.89 to 0.90, and the HR for the fourth quartile changed from 0.90 to 0.91.

## Discussion

### Main findings

Women with high adherence to a “main meal” frequency pattern, implying a regular intake of breakfast, lunch, and dinner, had a significantly lower risk of preterm delivery compared with women with low adherence to this pattern, even after adjustment for fiber as a proxy for a healthy diet. This independent association was seen with overall preterm delivery as well as in the group of overweight/obese women, and showed a similar trend in the subgroup of late preterm delivery. In the analysis of the glycemic properties, there were no significant associations between GI, GL, total carbohydrates, added sugar, or dietary fiber intake and preterm delivery.

### Our findings and those of other studies

To our knowledge, no other study has compared meal frequency patterns analyzed using principal component factor analysis in relation to pregnancy outcomes, e.g., preterm delivery. However, several other studies have demonstrated associations between meal frequency and preterm delivery. In one prospective study of more than 2000 pregnant women who were followed up using food frequency and meal frequency questionnaires, it was found that women who ate less than three main meals and more than two snacks daily during pregnancy had an 87% increased risk of preterm premature rupture of membranes followed by preterm delivery [38]. However, we found no association between meal frequency patterns and PPROM. On the other hand, it has been clinically shown that after the Yom Kippur Jewish fasting period, pregnant women have an increased risk of preterm labor [39]. A recent MoBa study found that women with high adherence to a Nordic diet, defined as eating regular meals throughout the day, together with an overall healthy eating pattern, had a significantly reduced risk of preterm delivery [9].

Although speculative, it is possible that women who fulfill their energy needs through regular main meals have a more stable plasma glucose response compared to “snack consumers” and women eating in the evening. We found that women who were considered overweight and/or obese, in particular, had a lower risk of preterm delivery when they ate according to a “main meal” pattern. Overweight subjects are typically more insulin resistant than are normal weight subjects [40]. Balanced plasma glucose levels are considered beneficial, especially in overweight pregnant women, as elevated glucose levels are correlated to an increased inflammatory response with increased levels of interleukins (IL), IL-1, IL-6, and TNF-α [41–44], such as have been observed in cases of preterm delivery [45].

In our study, we used a data-driven technique to extract meal patterns in the study cohort that explained more than half of the variance (52%). This method reduces the dimension of possible combinations of meals while retaining information about variance The association between high GL and late preterm delivery was no longer significant after Bonferroni correction. Therefore, we could not confirm the associations found in other studies. In a study on overweight and obese women, an association was found between high GL and increased risk of preterm delivery [23], and a different study found associations between dietary GI and preterm delivery [46]. It is possible that foods with a high GL may increase plasma glucose concentrations, as Scholl et al. [47] reported an increased risk of preterm delivery in non-diabetic women with higher plasma glucose levels. Furthermore, a previous study found that a high intake of sugar-sweetened beverages was associated with preterm delivery [48]. The reliability of GI values has been questioned, since values from the same test food could vary greatly both inter- and intra-individually. In one recently published study, it was found that the GI values from the same test food could vary as much as 20% within the same individual and 25% between individuals [49]. A high intake of sugar-sweetened beverages corresponds well to a high intake of energy from sugar [50]. However, it is more difficult to estimate the total amount of added sugar in foods since the sugar content of two similar food items can differ considerably, and these differences cannot be ascertained in an FFQ. On the other hand, there are not many differences between the sugar content of the various brands of sugar-sweetened drinks, and thus it is easier to obtain an accurate calculation. The association between a high intake of dietary fiber and a reduced risk of overall preterm delivery shown in younger women also did not persist after the Bonferroni correction.

Since we found no associations between the glycemic properties and preterm delivery in our analysis, and the reliability of the GI values was questionable, we chose not to include these as confounding factors in our analysis of the meal frequency patterns. However, in a separate analysis with separately presented data, we adjusted the “main meal” frequency pattern for dietary fiber intake as a proxy for an overall healthy lifestyle. In our previously published study [34], we found that women who chose an overall “healthy/prudent” diet comprising high amounts of dietary fiber had a significantly reduced risk of preterm delivery. We wanted to exclude that the “main meal” frequency pattern was not confounded by an overall healthy diet. The result showed that the “main meal” pattern was independently associated to a lower risk, as adjustments did not alter the results.

### Strengths and limitations

One of the strengths of this study is the large size of the study sample, which included women from all over Norway and all socioeconomic groups. The prospective study design is another important strength, since women were unaware of their pregnancy outcome when answering the FFQ. Furthermore, we had information about a number of covariates for which we were able to adjust. However, our study also has limitations. First, this was an observational study and conclusions about causality cannot be drawn. There is always a risk that the results are an effect of confounding factors instead of the exposure under investigation. However, we were able to adjust for previous preterm deliveries, which is one of the major risk factors of preterm delivery [51]. There is a participation rate of 40.6% in the ongoing MoBa cohort study, with participating women being generally better educated, older, and smoking less as compared to the general pregnant population of Norway [24]. Higher education levels and less smoking are associated with a generally healthier diet [52] [53], as well as a lower risk of preterm delivery [34]; however, both of these factors were adjusted for. Furthermore, in a study by Nilsen et al. [54] that focused on a potential self-selection bias in the MoBa cohort, it was shown that although the prevalence of both exposures and outcomes differed, the exposure-outcome associations regarding preterm delivery did not differ between the study cohort and the general pregnant Norwegian population. In this study, we chose to exclude women with all types of diabetes, since they are given dietary advice, especially with regard to regular meal frequency. Women with diabetes have an increased risk of preterm delivery [55] and induction is more common in that population because large-for-gestational age (LGA) is one of the most common complications [52]. Therefore, our results are not generalizable for women with any type of diabetes. The FFQ used in our study has been extensively validated against a four-day food diary and several biomarkers of intake, and it has been shown to be a valuable tool for ranking pregnant women according to high and low intakes of energy and nutrients [28]. However, the FFQ has not been validated with regard to the calculations of dietary GI and GL. Generally, the use of FFQs to study dietary GI and GL has been questioned because of limitations in the number of questions asked, the aggregation of several food items in one question, and a lack of day-to-day variation. For calculations of GI and GL, the focus is on carbohydrate-rich food items, and the MoBa FFQ included six aggregated questions for different types of bread, buns, crisp bread, and crackers, with a single GI value for each question. The lack of associations between daily dietary GI and GL and preterm delivery might be due the fact that the FFQ was not detailed enough to represent the true GI and GL of the diet [56]. Studying dietary habits is always challenging because all dietary assessment methods are prone to misreporting. For example, in this FFQ, women were asked to report their daily intake of food and beverages during the first four months of pregnancy, which carries a risk of recall bias. As in the case of added sugar [57], the calculations of total daily GI and GL are also blunt estimates of differences in postprandial blood sugar fluctuations and entail many assumptions [58].

## Conclusions

In summary, we found that women adhering to regular main meals had a significantly reduced risk of preterm delivery. This meal frequency pattern included eating breakfast, lunch, and dinner on a regular basis, with a median of one additional snack per day, among those with the strongest adherence to the pattern. No significant associations were seen between GI, GL, total carbohydrate, added sugar, or dietary fiber intake in relation to preterm delivery. These findings highlight the potential importance of meal frequency patterns during pregnancy for infant health.

## Supporting information

S1 Fig Scree plot for identification of meal frequency patterns by principal component factor analysis of eight meals per day (breakfast, morning snack, lunch, afternoon snack, dinner, evening snack, supper, night meal) in 65,487 pregnant women in the Norwegian Mother and Child Cohort Study (MoBa). (TIFF)

S1 Table Pattern scores of maternal meal frequency patterns in relation to maternal characteristics in 65,487 women in the Norwegian Mother and Child Cohort Study (MoBa). (DOCX)

S2 Table Glycemic properties and daily intake of total carbohydrates, added sugar and dietary fiber and by maternal characteristics in 66,000 women in the Norwegian Mother and Child Cohort Study (MoBa). (DOCX)

S3 Table Associations between glycemic properties and preterm delivery in 66,000 pregnant women in the Norwegian Mother and Child Cohort Study (MoBa) (DOCX)

## Abbreviations

CI: confidence interval
FFQ: food frequency questionnaire
GI: glycemic index
GL: glycemic load
HR: hazard ratio
MoBa: the Norwegian Mother and Child Cohort Study
PTD: preterm delivery
Q: questionnaire
