# Supplementary figures and images for: Meal frequency patterns and glycemic properties of maternal diet in relation to preterm delivery: Results from a large prospective cohort study
Source: PLoS One. 2017 Mar 1;12(3):e0172896. doi: 10.1371/journal.pone.0172896 (PMC5332093; doi:10.1371/journal.pone.0172896)

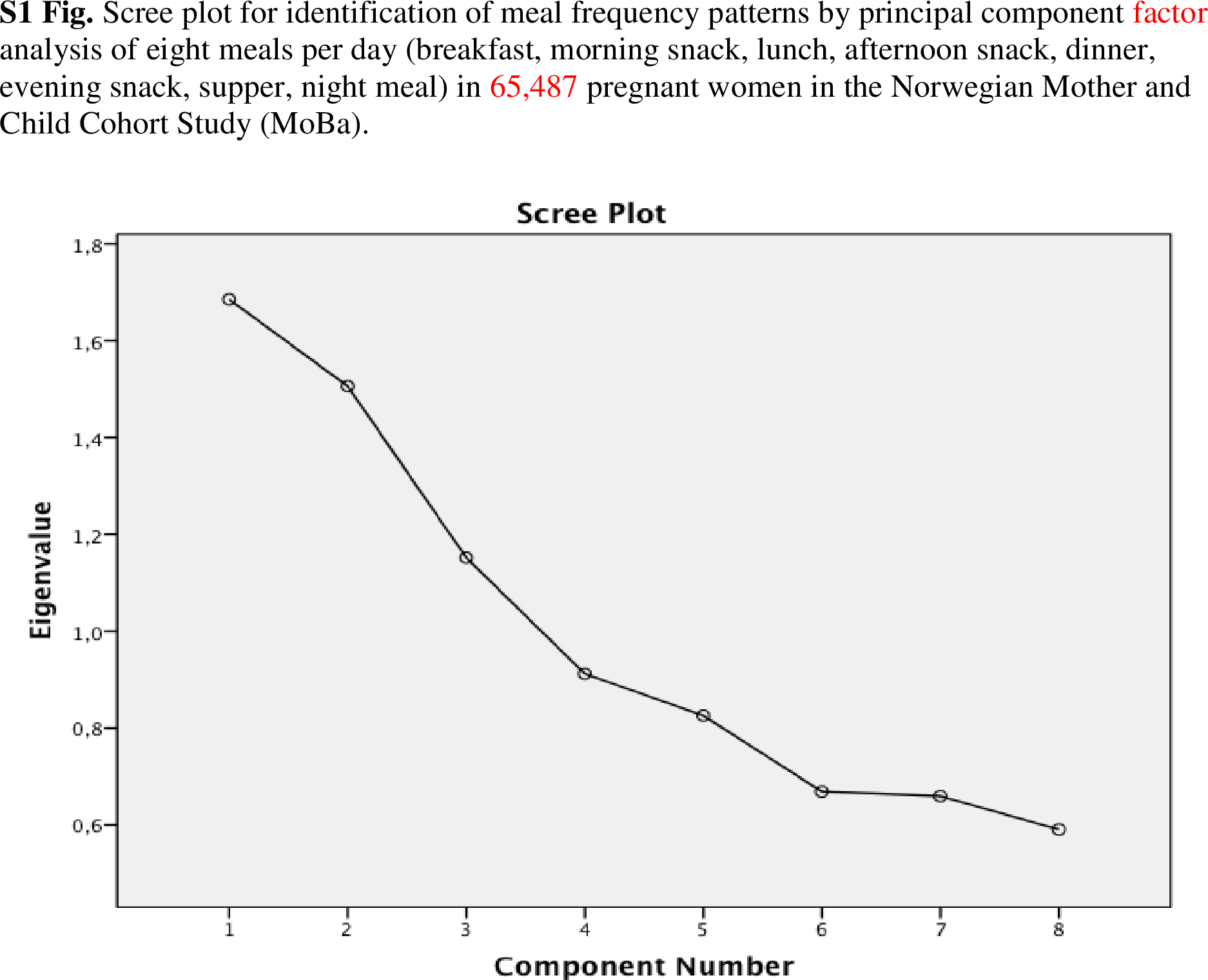

Supplement: S1 Fig — (TIFF) [file pone.0172896.s001.tiff]
