# Supplementary material for: Meal frequency patterns and glycemic properties of maternal diet in relation to preterm delivery: Results from a large prospective cohort study
Source: PLoS One. 2017 Mar 1;12(3):e0172896. doi: 10.1371/journal.pone.0172896 (PMC5332093; doi:10.1371/journal.pone.0172896)
Supplement: S1 Table — (DOCX) [file pone.0172896.s002.docx]

### S1 Table. Pattern scores of maternal meal frequency patterns in relation to maternal characteristics in 65,487 women in the Norwegian Mother and Child Cohort Study (MoBa).

|  | **Women, n (%)** | **Snack meal pattern^1^** | **Main meal pattern^1^** | **Evening meal**  **Pattern^1^** |
| --- | --- | --- | --- | --- |
| Maternal age at delivery, *y* |  |  |  |  |
| < 35 | 54,381 (83.2) | 0.004±0.996 | -0.030±1.019 | 0.016±0.996 |
| ≥ 35 | 11,106 (16.8) | -0.022±1.019 | 0.148±0.889 | -0.080±1.015 |
| *p value*^2^ |  | 0.012 | <0.001 | <0.001 |
| Maternal education, *y* |  |  |  |  |
| ≤ 12 | 20,255 (29.9) | -0.063±1.050 | -0.326±1.195 | 0.172±1.034 |
| 13-16 | 27,267 (41.6) | -0.008±0.971 | 0.104±0.881 | -0.013±0.958 |
| ≥ 17 |  | 0.084±0.972 | 0.240±0.789 | -0.188±0.984 |
| Missing | 16,594 (26.4) | 0.065±1.055 | -0.148±1.109 | -0.002±1.057 |
| *p value*^3^ | 1,371 (2.1) | <0.001 | <0.001 | <0.001 |
| History PTD |  |  |  |  |
| No | 63,164 (96.7) | 0.006±1.000 | -0.001±1.000 | -0.004±1.001 |
| Yes | 2,323 (3.3) | -0.165±1.000 | 0.037±1.007 | 0.106±0.974 |
| *p value*^3^ |  | <0.001 | <0.001 | <0.001 |
| Parity |  |  |  |  |
| Nulliparous | 33,968 (54.5) | 0.112±0.995 | -0.071±1.022 | -0.038±1.020 |
| Parous | 31,519 (45.5) | -0.120±0.992 | 0.077±0.969 | 0.040 ±0.096 |
| *p value*^2^ |  | <0.001 | <0.001 | <0.001 |
| BMI^4^, *kg/m²* |  |  |  |  |
| < 18.5 | 2,017 (3.3) | 0.210±1.084 | -0.124±1.126 | 0.237±1.120 |
| 18.5-24.9 | 42,398 (66.0) | 0.043±1.004 | 0.053±0.963 | -0.005±1.001 |
| 25-29.9 | 13,643 (20.2) | -0.070±0.974 | -0.042±1.012 | -0.022±0.977 |
| ≥ 30 | 5,769 (8.0) | -0.201±0.954 | -0.190±1.088 | -0.007±0.982 |
| missing | 1,660 (2.4) | -0.081±1.026 | -0.189±1.201 | 0.034±1.040 |
| *p value*^3^ |  | <0.001 | <0.001 | <0.001 |
| Smoking |  |  |  |  |
| No | 59,871 (92.0) | 0.012±0.995 | 0.052±0.951 | -0.0218±0.990 |
| Occasional | 1,725 (2.5) | -0.118±0.998 | -0.411±1.173 | 0.137±1.033 |
| Daily | 3,520 (5.0) | -0.139±1.071 | -0.678±1.347 | 0.297±1.099 |
| missing | 371 (0.5) | -0.051±1.010 | -0.102±1.146 | 0.054±1.121 |
| *p value*^3^ |  | <0.001 | <0.001 | <0.001 |
| Marital status |  |  |  |  |
| Cohabiting | 62,964 (96.1) | -0.002±0.995 | 0.021±0.980 | -0.010±0.994 |
| Single | 2,523 (3.9) | 0.048±1.105 | -0.526±1.313 | 0.252±1.115 |
| *p value*^2^ |  | 0.378 | <0.001 | <0.001 |
| Income in NOK^5^ |  |  |  |  |
| Either <300´ | 18,182 (27.1) | -0.046±1.029 | -0.215±1.142 | 0.179±01.012 |
| Either >300´ | 26,866 (40.7) | -0.018±1.001 | 0.030±0.962 | 0.015±0.982 |
| Both >300´ | 18,572 (28,4) | 0.071±0.956 | 0.202±0.804 | -0.222±0.966 |
| missing | 1,867 (2.8) | -0.004±1.096 | -0.349±1.289 | 0.253±1.048 |
| *p value*^3^ |  | <0.001 | <0.001 | <0.001 |
| Total energy intake, *kJ* |  |  |  |  |
| Quartile 1 | 16,307 (24.9) | -0.175±0.944 | -0.150±1.092 | -0.288±0.969 |
| Quartile 2 | 16,385 (25) | -0.059±0.970 | 0.070±0.915 | -0.106±0.962 |
| Quartile 3 | 16,402 (25) | 0.024±0.990 | 0.099±0.923 | 0.075±0.953 |
| Quartile 4 | 16,393 (25) | 0.209±1.057 | -0.019±1.034 | 0.317±1.014 |
| *p value*^2^ |  | <0.001 | <0.001 | <0.001 |
| Nausea^6^ |  |  |  |  |
| No | 43,338 (65.6) | -0.026±0.982 | 0.048±0.951 | -0.052±0.987 |
| Yes | 22,149 (34.4) | 0.051±1.032 | -0.094±1.083 | 0.102±1.018 |
| *p value*^2^ |  | <0.001 | <0.001 | <0.001 |

^1^ Mean and standard deviation of continues meal frequency pattern scores. Positive scores denote high adherence to a pattern and negative scores denote low adherence to a pattern.

^2^ANOVA

^3^ Non-parametric Mann-Whitney test (two groups) or non-parametric test Kruskal-Wallis (more than two groups).

^4^ Pre-pregnancy

^5^ Total household income

^6^ Experienced nausea during first trimester
