# Supplementary material for: Meal frequency patterns and glycemic properties of maternal diet in relation to preterm delivery: Results from a large prospective cohort study
Source: PLoS One. 2017 Mar 1;12(3):e0172896. doi: 10.1371/journal.pone.0172896 (PMC5332093; doi:10.1371/journal.pone.0172896)
Supplement: S2 Table — (DOCX) [file pone.0172896.s003.docx]

**S2 Table.** Glycemic properties and daily intake of total carbohydrates, added sugar and dietary fiber and by maternal characteristics in 66,000 women in the Norwegian Mother and Child Cohort Study (MoBa).

|  | Women, n (%) | Glycemic  index^1^ | Glycemic  load² | Total  Carbohydrates³ | Added sugar ³ | Fiber³ |
| --- | --- | --- | --- | --- | --- | --- |
| Maternal age at delivery, *y* |  |  |  |  |  |  |
| < 35 | 54,792 (83.0) | 55.1±11 | 166±51 | 311±94 | 64±40 | 31±11 |
| ≥ 35 |  | 56.0±10 | 162±48 | 303±88 | 56±34 | 32±11 |
| *p value*^4^ | 11,208 (17,0) | <0.001 | <0.001 | <0.001 | <0.001 | <0.001 |
| Maternal education, *y* |  |  |  |  |  |  |
| ≤ 12 | 20,472 (31.0) | 55.2±12 | 169±56 | 321±105 | 70±48 | 31±12 |
| 13-16 | 27,423 (41.6) | 55.5±10 | 164±48 | 306±88 | 60±35 | 31±10 |
| ≥ 17 | 16,704 (25.3) | 54.9±10 | 162±46 | 303±84 | 57±31 | 32±10 |
| missing | 1,401 (2.1) | 54.7±11 | 162±53 | 305±98 | 62±40 | 30±11 |
| *p value*^5^ |  | <0.001 | <0.001 | <0.001 | <0.001 | <0.001 |
| History PTD |  |  |  |  |  |  |
| No | 63,667 (96.5) | 55.2±10 | 165±50 | 310±93 | 62±39 | 31±11 |
| Yes | 2,333 (3.5) | 55.9±10 | 169±52 | 317±97 | 65±42 | 32±11 |
| *p value*^5^ |  | 0.003 | <0.001 | 0.001 | 0.002 | 0.052 |
| Parity |  |  |  |  |  |  |
| Nulliparous | 34,217 (51.8) | 54.6±10 | 163±51 | 306±94 | 62±39 | 31±11 |
| Parous | 31,783 (48.2) | 55.9±10 | 167±50 | 313±92 | 63±40 | 31±11 |
| *p value*^5^ |  | <0.001 | <0.001 | <0.001 | <0.001 | <0.001 |
| BMI, *kg/m²* |  |  |  |  |  |  |
| < 18.5 | 2029 (3.1) | 55.5±11 | 177±57 | 332±106 | 73±50 | 32±12 |
| 18.5-24.9 | 42,704 (64.7) | 55.2±10 | 166±50 | 312±92 | 62±38 | 31±11 |
| 25-29.9 | 13,754 (20.8) | 55.2±11 | 162±50 | 304±92 | 61±38 | 30±11 |
| ≥ 30 | 5,824 (8.8) | 55.3±11 | 159±51 | 300±95 | 61±42 | 30±11 |
| missing | 1,689 (2.6) | 54.2±11 | 165±53 | 313±100 | 65±45 | 31±11 |
| *p value*^5^ |  | 0.001 | <0.001 | <0.001 | <0.001 | <0.001 |
| Smoking |  |  |  |  |  |  |
| No | 60,321 (91.4) | 55.2±10 | 164±50 | 308±91 | 61±37 | 31±11 |
| Occasional | 1,748 (2.6) | 54.7±12 | 169±57 | 324±107 | 75±49 | 31±12 |
| Daily | 3,553 (5.4) | 55.1±12 | 172±59 | 334±111 | 83±58 | 30±11 |
| missing | 378 (0.6) | 55.1±11 | 166±56 | 316±106 | 66±49 | 32±12 |
| *p value*^5^ |  | <0.001 | <0.001 | <0.001 | <0.001 | <0.001 |
| Marital status |  |  |  |  |  |  |
| Cohabiting | 63,446 (96.1) | 55.2±10 | 165±50 | 309±92 | 62±39 | 31±11 |
| Single | 2,554 (3.9) | 54.6±12 | 171±59 | 325±110 | 72±50 | 31±12 |
| *p value*^5^ |  | <0.001 | <0.001 | <0.001 | <0.001 | 0.755 |
| Income in NOK |  |  |  |  |  |  |
| Either <300´ | 18,352 (27.8) | 55.2±11 | 171±54 | 321±100 | 68±44 | 31±11 |
| Either >300´ | 27,051 (41.0) | 55.4±10 | 166±50 | 311±92 | 62±39 | 31±11 |
| Both >300´ | 18,698 (28,3) | 54.9±10 | 157±46 | 295±83 | 56±31 | 31±10 |
| missing |  | 54.9±12 | 172±58 | 327±110 | 70±51 | 32±13 |
| *p value*^5^ | 1,899 (2.9) | <0.001 | <0.001 | <0.001 | <0.001 | 0.001 |
| Total energy intake, *kJ* |  |  |  |  |  |  |
| Quartile 1 | 16,500 (25) | 52.8±11 | 111±21 | 210±34 | 38±19 | 22±6 |
| Quartile 2 | 16,500 (25) | 55.3±10 | 146±17 | 273±27 | 51±23 | 28±6 |
| Quartile 3 | 16,500 (25) | 56.1±10 | 174±20 | 325±33 | 65±29 | 33±7 |
| Quartile 4 | 16,500 (25) | 56.7±11 | 229±41 | 430±75 | 97±50 | 41±11 |
| *p value*^4^ |  | <0.001 | <0.001 | <0.001 | <0.001 | 0.001 |

^1^ Glycemic index, defined as the incremental area under the two-hour blood glucose response curve

² Glycemic load = carbohydrates (g) in a serving size x GI/100

³ Daily intake as mean and standard deviation

^4^ANOVA

^5^Non-parametric Mann-Whitney test (two groups) or non-parametric test Kruskal-Wallis (more than two groups)

^6^ Pre-pregnancy

^7^ Total household income
