# Supplementary material for: Meal frequency patterns and glycemic properties of maternal diet in relation to preterm delivery: Results from a large prospective cohort study
Source: PLoS One. 2017 Mar 1;12(3):e0172896. doi: 10.1371/journal.pone.0172896 (PMC5332093; doi:10.1371/journal.pone.0172896)
Supplement: S3 Table — (DOCX) [file pone.0172896.s004.docx]

### S3 Table. Associations between glycemic properties and preterm delivery in 66,000 pregnant women in the Norwegian Mother and Child Cohort Study (MoBa)

| **Component** | **PTD**  **n (%^1^)** | **Model 1²**  **HR (95% CI)** | **Model 2³**  **HR (95% CI)** | **Model 3^4^**  **HR (95% CI)** |
| --- | --- | --- | --- | --- |
| All | 3,502 (5.3) |  |  |  |
| Glycemic index |  |  |  |  |
| Quartile 1 | 916 (5.6) | 1 | 1 | 1 |
| Quartile 2 | 842 (5.1) | 0.92 (0.83, 1.01) | 0.95 (0.86, 1.04) | 0.95 (0.86, 1.04) |
| Quartile 3 | 895 (5.4) | 0.98 (0.89, 1.07) | 1.01 (0.92, 1.11) | 1.02 (0.93, 1.12) |
| Quartile 4 | 852 (5.2) | 0.93 (0.84, 1.02) | 0.94 (0.86, 1.04) | 0.96 (0.87, 1.05) |
| *p* for trend^5^ |  | *0.116* | *0.479* | *0.697* |
| Glycemic load |  |  |  |  |
| Quartile 1 | 847 (5.1) | 1 | 1 | 1 |
| Quartile 2 | 876 (5.3) | 1.04 (0.94, 1.14) | 1.07 (0.97, 1.19) | 1.08 (0.97, 1.21) |
| Quartile 3 | 861 (5.2) | 1.02 (0.92, 1.12) | 1.07 (0.94, 1.21) | 1.10 (0.96, 1.26) |
| Quartile 4 | 921 (5.6) | 1.09 (0.99, 1.20) | 1.11 (0.93, 1.32) | 1.18 (0.98, 1.42) |
| *p* for trend |  | *0.266* | *0.272* | *0.102* |
| Carbohydrates |  |  |  |  |
| Quartile 1 | 836 (5.1) | 1 | 1 | 1 |
| Quartile 2 | 890 (5.4) | 1.07 (0.97, 1.17) | 1.10 (0.99, 1.23) | 1.11 (0.99, 1.25) |
| Quartile 3 | 861 (5.2) | 1.03 (0.94, 1.13) | 1.07 (0.94, 1.22) | 1.11 (0.96, 1.27) |
| Quartile 4 | 918 (5.6) | 1.10 (1.00, 1.21) | 1.11 (0.93, 1.34) | 1.18 (0.97, 1.43) |
| *p* for trend |  | *0.097* | *0.322* | *0.141* |
| Added sugar |  |  |  |  |
| Quartile 1 | 883 (1.3) | 1 | 1 | 1 |
| Quartile 2 | 842 (1.3) | 0.95 (0.87, 1.05) | 0.96 (0.87, 1.06) | 0.96 (0.87, 1.05) |
| Quartile 3 | 853 (1.3) | 0.97 (0.88, 1.06) | 0.97 (0.87, 1.07) | 0.95 (0.86, 1.05) |
| Quartile 4 | 927 (1.4) | 1.05 (0.96, 1.15) | 0.99 (0.88, 1.11) | 0.96 (0.85, 1.08) |
| *p* for trend |  | *0.261* | *0.854* | *0.467* |
| Dietary fiber |  |  |  |  |
| Quartile 1 | 888 (5.4) | 1 | 1 |  |
| Quartile 2 | 886 (5.4) | 1.00 (0.91, 1.10) | 1.01 (0.91, 1.12) |  |
| Quartile 3 | 877 (5.3) | 0.99 (0.90, 1.08) | 0.98 (0.88, 1.08) |  |
| Quartile 4 | 854 (5.2) | 0.96 (0.87, 1.06) | 0.90 (0.79, 1.02) |  |
| *p* for trend |  | *0.381* | *0.097* |  |

^1^ Percentage of preterm delivery in each quartile

² Unadjusted hazard ratio

³ Hazard ratios adjusted for non-dietary covariates: maternal age, pre pregnancy BMI, height, parity, total energy intake, maternal education, marital status, smoking, income and previously preterm delivery

^4^ Hazard ratios additionally adjusted for dietary fiber

^5^ *p* values for linear trend were obtained by incorporating the variable as a linear term in Cox regression models
